# Managing cassava growth on nutrient poor soils under different water stress conditions

**DOI:** 10.1016/j.heliyon.2021.e07331

**Published:** 2021-06-16

**Authors:** Matema L.E. Imakumbili, Ernest Semu, Johnson M.R. Semoka, Adebayo Abass, Geoffrey Mkamilo

**Affiliations:** a Department of Soils and Geological Sciences, Sokoine University of Agriculture, Morogoro, Tanzania; b The International Institute of Tropical Agriculture, Dar es Salaam, Tanzania; c Roots and Tubers Department, Naliendele Agricultural Research Institute, Mtwara, Tanzania

**Keywords:** NPK fertilizers, Drought stress, New leaf formation, Crop water use, Plant growth, Leaf size, Nutrient supply, Climate change adaptation, Pot experiment, Three-way interactions

## Abstract

Nitrogen (N), phosphorus (P) and potassium (K) fertiliser application, was able to counteract growth reductions, in cassava cultivated on nutrient poor soils, under one water stress condition. It however remains to be seen, whether N, P and K fertiliser application, would produce similar results, across different water stress conditions. A study was therefore conducted to determine how N, P and K fertiliser application, would influence cassava growth on nutrient poor soils, under various water stress conditions. Effects on new leaf formation and leaf size were also investigated. The study was a 2×3×4 factorial pot experiment, in a randomised complete block design. It included: two cassava varieties, three water stress levels and four fertiliser treatments. The water stress levels kept some plants watered at field capacities of 30% (severe water stress), 60% (mild water stress) and 100% (zero water stress). The fertiliser treatments consisted of a control (no fertiliser), a sole K fertiliser treatment (25 mg K/kg), a moderate N, P and K fertiliser treatment (25 mg N + 5 mg P + 25 mg K/kg) and a high N, P and K fertiliser treatment (50 mg N + 13 mg P + 50 mg K/kg). All data were analysed using the analysis of variance. Cassava growth was assessed by monitoring changes in the dry shoot mass of cassava plants. High and moderate N, P and K fertiliser application, produced cassava plants with higher and similar dry shoot masses, under mild water stress (10.5 g/plant, SE = 0.6 and 9.0 g/plant, SE = 0.6, respectively). High N, P and K fertiliser application, however gave cassava the highest dry shoot mass, under severe water stress (7.9 g/plant, SE = 0.4). Relatively high cassava growth was consistently achieved with high N, P and K fertiliser application, across all water stress conditions.

## 1 Introduction

With climate change either reducing rainfall amounts or causing prolonged dry spells, in several parts of the world [56], the efficient use of the often limited available water, is becoming more and more critical, for lessening the negative impacts of low and erratic rainfall on crop growth and yields. The efficient use of water in crop production is better known as agronomic water use efficiency or simply as water use efficiency (WUE). It is defined as the amount of biomass produced compared to the amount of water lost through evapotranspiration [1]. Water use efficiency, therefore functions to ensure the maximum and non-wasteful use of water resources, during crop production. Water use efficiency can be improved: (i) by planting crops that are better at maximising transpiration, (ii) or by using crop management practices that are able to reduce evaporation from crop fields (iii) or by the use of practices that increase the amount of water supplied to growing crops [2].

A number of crops like cassava (*Manihot esculenta* Crantz), cowpeas (*Vigna unguiculata*) and sorghum (*Sorghum bicolor*) are known to be drought tolerant. The crops are therefore better adapted to water stress. A number of drought tolerant mechanisms enable them to maximise transpiration and to thereby utilise water more efficiently. Drought tolerant mechanisms enable crops to survive and/or remain productive during periods of prolonged water stress. Focusing on cassava, one of its important water conserving mechanisms, during periods of prolonged water stress, is leaf canopy reduction [3]. With a reduced leaf canopy, cassava is able to decrease its leaf area and to thereby reduce its transpiration rate to match the available supply of water. To reduce its leaf canopy size, cassava restricts new leaf formation, produces smaller sized leaves and sheds-off older leaves [4]. This however results in reduced crop growth, lower biomass production and in reduced root yields [5]. Water conservation is hence achieved, but at the expense of crop productivity.

Under water limited conditions, the application of nitrogen (N) fertilisers to soils lacking N, was shown to improve maize (*Zea mays* L.) yields [6], while the application of phosphorous (P) fertilisers to soils lacking P, was reported to increase the yields of soybean (*Glycine max* (L.) Merr.) [7]. An improved supply of P encourages root development, which in turn leads to greater water uptake by plants [7, 8]. Rapid increments in plant foliage, on the hand occur, with an improved supply of N; resulting in increased water loss through higher transpiration [9]. The water loss is however counteracted by reduced evaporation from soil surfaces, due to improved shading, from expanded leaf canopies. Application of P fertilisers therefore functions to increase water supply to plants, while application of N fertilisers helps to conserve soil moisture, while maintaining the photosynthetic capacity of plants. Increased WUE is hence achieved in all cases. Application of potassium (K) fertilisers has also been reported to enhance WUE in plants [10, 11]. Increments in plant growth, due to improved WUE, were reportedly observed with the application of K fertilisers [11]. Potassium particularly plays an important role in mitigating many forms of plant stress, including water stress [12]. Application of N, P and K fertilisers, can hence be beneficial for counteracting reductions in the growth and yields of many crops, under water limited conditions.

Application of N, P and K fertilisers, was able to counteract reductions in the production of dry matter, in cassava cultivated on nutrient poor soils, under one water stress condition (level) [13]. While the application of N, P and K fertilisers, was shown to relatively maintain high cassava growth, on nutrient poor soils, under one water stress condition, it remains to be seen whether this trend can be maintained across all water stress conditions. This is important to know as water stress can vary from severe to mild. Some cassava growing areas may moreover be prone to either severe or mild water stress, or indeed to both of these forms of water stress. Knowing how N, P and K fertiliser application, would affect cassava growth on nutrient poor soils, across different water stress conditions, is hence essential. This knowledge would ultimately bring some understanding to how water stress affects the productivity of cassava, on soils with varied fertility.

This study was carried out to investigate whether N, P and K fertiliser application, can help to maintain cassava growth, on nutrient poor soils, across different water stress conditions. The dry shoot mass of cassava plants (stems and leaves), was used to estimate changes in cassava growth. Losses or gains in the shoot biomass of cassava (and thus of its shoot mass), are reflective of expected changes on fresh root yields and on the general growth of cassava plants [14]. The hypothesis tested was therefore that *the application of N, P and K fertilisers, on nutrient poor soils, does not influence the dry shoot mass of cassava plants, under different water stress conditions*. Effects on new leaf formation and on leaf size, were additionally investigated to reveal the changes that occur to the leaf canopy size of cassava, in order to achieve the reduced losses in cassava growth. The second hypothesis tested was therefore that *the application of N, P and K fertilisers, on nutrient poor soils, does not influence new leaf formation and the leaf size of cassava plants, under different water stress conditions.*

## 2 Materials and methods

### 2.1 Study location

The study was carried out as a pot experiment. It was conducted at Sokoine University of Agriculture (SUA) (S 6°51′13″, E 37°39′26″), in Morogoro district, in Tanzania. The experiment was conducted under screen-house conditions, over a period of 90 days, from the 24^th^ October 2015 to 25^th^ January 2016.

### 2.2 Soil collection and preparation for potting

The soil used as a potting medium was collected from Soga village (S 6°49′54″, E 38°51′49″), in Kibaha district, in Tanzania. Soils in Kibaha district are predominantly Ferralic Cambisols; they are sandy in nature and have an inherently low soil fertility [15, 16]. Ten different points were selected on a field prior to soil collection [17, 18]. Surface litter was then removed from a sufficiently wide area (about 1.0–1.5 m^2^), around each selected point, before collecting top soil from a depth of 0–20 cm. The collected soil was packed into polypropylene sacks and transported to SUA, where it was bulked into one composite heap. The soil was then thoroughly mixed before passing it through an 8 mm sieve to facilitate drying and to remove large pieces of debris. The soil was then left to dry for more than 2 weeks, during which it was consistently turned over. When the soil was sufficiently dry, its moisture content (0.15%) was determined using the gravimetric method [19]. The soils moisture content was used to calculate the mass of air-dry soil (5.0075 kg), needed to give 5 kg of oven-dry soil. Each pot was filled with air-dry soil with a mass equivalent to 5 kg of oven-dry soil. Uniform plastic pots, with a diameter of 22 cm and a depth of 17 cm, were used in the experiment. Each pot was watered to field capacity, one day before planting and was left to saturate overnight.

### 2.3 Experimental design and treatments

The pot experiment was a 2×3×4 factorial experiment, in a randomised complete block design (RCBD). The treatments consisted of two cassava varieties, three water stress levels and four different fertiliser combinations and amounts. The blocks were replicated six times. Only five blocks were however considered in the analysis, as one block was removed, due to a few incorrectly labelled pots. The treatments used are described in greater detail below.

#### 2.3.1 Cassava varieties

The two cassava varieties used in the pot experiment, included a local cassava variety called *Salanga* and an improved cassava variety called *Kiroba*. *Salanga* was collected from Kitangari village (S 10°39′01″, E 39°20′01″), in Newala district, in Tanzania, while *Kiroba* was collected from Naliendele Agricultural Research Institute (NARI) (S 10°21′22″, E 40°09′59″), in Mtwara district, in Tanzania. The stem cuttings of the two varieties were collected from visibly healthy mature cassava plants, of the same age. Previously rooted cassava plantlets were used to establish the pot experiment [20, 21]. This was because, unlike 20–30 cm long mature cassava stem cuttings, rooted cassava plantlets have depleted nutrient reserves. Responses to changes in soil nutrient supply can thus be quickly seen in experiments, when rooted cassava plantlets are used. Rapid cassava stem cutting multiplication, was used to produce the rooted cassava plantlets [22]. Following with these methods, the collected cassava stem cuttings, were cut into several small 10 cm long stem cuttings, before being densely planted, at a spacing of 10 cm × 10 cm, in nursery beds with nutrient poor soil. The cuttings were then left to sprout until their shoots were 15 cm long. The shoots were then cut off and rooted in distilled water, to produce the rooted cassava plantlets. It took the shoots about one month to sufficiently root. The experiment was established by planting one rooted cassava plantlet per pot.

#### 2.3.2 Water stress treatments

All pots were kept well-watered (zero stressed) at 100% field capacity (FC), during the first 70 days after planting (DAP). Field capacity (100% FC) is described as the soil moisture content of a fully wetted soil, right after drainage; the water and air contents of soils are considered ideal for crop growth at field capacity [23]. The water stress treatments were only begun at 71 DAP and were maintained for 20 days (until 90 DAP). They included water stress levels that kept plants watered to (i) 100% FC (zero stress or well-watered); to (ii) 60% FC (mild water stress); and lastly to (iii) 30% FC (severe water stress) [24, 25, 26]. Field capacities of 30% and 60% were selected, because they closely depict soil moisture contents that are respectively about a quarter and about half the soil moisture content at 100% FC. A FC of 25% (exactly a quarter of 100% FC) could not be used to represent severe water stress, as the plants were unable to survive under this soil moisture content, as the soils were sandy. A FC of 30% was hence selected. This also meant that 50% FC (exactly half of 100% FC) could not be used to represent mild water stress. It was instead adjusted upwards to 60% FC, to create a reasonable difference between itself and the soil moisture contents used to create severe water stress and well-watered conditions.

After 70 DAP, pots under the well-watered treatment, continued to be brought to 100% FC, every day. Pots under the mild and severe water stress treatments, were however left to lose water, until they were slightly below their required FC levels. Once the pots had attained a slightly lower soil moisture content than either 60% or 30% FC, they were re-watered and brought to their required moisture contents. The pots continued to be maintained at their required soil moisture contents, with daily replacements of lost water, throughout the rest of the water stress period. The amount of water needed to bring the soil in each pot, to its respective moisture content, was determined by weighing [24, 25, 27]. All pots were re-watered at around 07:00 h each morning.

The amount of water needed to bring the potting soil to 100% FC was determined by filling three identical transparent 2 L containers, with some of the air-dry potting soil [28]. Water was then evenly poured over the soil surface, until the soil was fully wet. The containers were then immediately covered with plastic and placed in a cool area, away from sunlight, for 3 days [23]. This was done to allow the excess water to completely drain and also to let the soil to fully saturate the soil, to 100% FC. The containers had small holes at the bottom to allow excess water to drain out. Covering the containers prevented water loss by evaporation and ensured that the top layers of the soil also remained fully saturated. After 3 days, a 100 g sample of wet soil was collected from each container. The wet soil was taken from a 5–10 cm depth below the soil surface. The moisture content of the wet soil was then determined using the gravimetric method. The mean moisture content of the three replicates was used to represent the moisture content of the potting soil at 100% FC (18.67%).

Since air-dry soil was used to fill the pots in the pot experiment, the amount of water needed to bring its oven-dry equivalent mass to 100% FC, had to take into account the moisture already contained in the air-dry soil (18.67%–0.15% = 18.52%) (Eq. 1). The value of 18.52%, however represents the amount (mass) of water needed to bring a 1 g mass of oven-dry soil to 100% FC (0.1852 g/g) [19]. The mass of water needed to bring 5 kg (5000 g) of oven-dry soil to 100% FC, was hence calculated using Eq. (2); and it was found to be 926 g. Reporting the water required to bring the soil to FC as a mass, makes it easier to know the expected mass of pots after additions of water. Although the pots were weighed to determine the mass of water needed to be added, the water was however added in volume form. The mass of water added was thus always converted to its volume equivalent (mass ⁄ density), taking into consideration the density of water (1 g/cm^3^) [29]. The volume of water needed to bring the soil in pots to 60% and 30% FC, was calculated by determining the volume of water respectively equivalent to 60% (556 cm^3^) and 30% (278 cm^3^) of 926 cm^3^ (the volume of water needed to bring 5 kg of oven-dry soil to 100% FC).

(1) Amount of water needed to bring soil to FC(%)=moisture content at FC(%)−moisture content of air dry soil(%)

(2) $$\text{Mass }\;100\text{\% }\left(g\right)=\text{moisture }100\text{\% }\left(g/g\right)\times \text{oven }\left(g\right)$$

The amount of water replaced in each pot took into account the mass of the pot, the mass of soil and the mass of the plant [24]. Changes in the mass of the plant over time, were also taken into consideration using Eqs. (3) and (4) [24].

(3) $$\text{Total mass of pot at }t_{x}\left(g\right)=\left(\text{mass of pot}+\text{mass of plant at }t_{x}+\text{mass of oven dry soil}\right)g+\left(\text{mass of soil moisture}\right)g$$

where; *t*_*x*_ is the time period at which measurements are taken (*x* = 0, 1, 2, 3 … days after planting).

(4) Mass of water needed to be added (g)=total mass of pot at X% FC (g)−current total mass of pot (g)

where; X% is 100%, 60% or 30% field capacity (FC).

#### 2.3.3 Fertiliser treatments

The fertiliser treatments consisted of a control (no fertiliser applied), a sole K fertiliser treatment, a moderate N, P and K fertiliser treatment and a high N, P and K fertiliser treatment (Table 1). The sole K fertiliser treatment was included, given the importance of K for increased cassava growth and root yields [30, 31, 32]. The highest N, P and K fertiliser treatment was closely equivalent to the general recommended N, P and K fertiliser rate, for cassava grown in the field (100 kg N + 22 kg P + 83 kg K/ha or 100 kg N + 50 kg P_2_O_5_ + 100 kg K_2_O/ha) [33]. The moderate N, P and K fertiliser treatment, was on the other hand about half the general recommended N, P and K fertiliser rate, for cassava grown in the field (Table 1).

**Table 1:** Fertiliser treatments used in the pot experiment, together with their equivalent field-based fertiliser rates.

Fertiliser treatment	Pot-based fertiliser rates‡			Field-based fertiliser rates†		
	N	P	K	N	P	K
	(mg/kg)	(mg/kg)	(mg/kg)	(kg/ha)	(kg/ha)	(kg/ha)
Control	0	0	0	0	0	0
Sole K	0	0	25	0	0	50
Moderate N, P and K	25	5	25	50	10	50
High N, P and K	50	13	50	100	25	100

† The equivalent field-based N, P and K fertiliser rates are indicated as kilograms of nutrients added per hectare (kg/ha).

‡ The pot-based N, P and K fertiliser rates are indicated as milligrams of nutrients applied per kilogram of oven-dry soil (mg/kg).

The fertilisers urea (CO(NH_2_)_2_), triple super phosphate (TSP or Ca(H_2_PO_4_)_2_.H_2_O) and muriate of potash (MOP or KCl), were used to respectively supply N, P and K. All the MOP and the TSP were added to the potting soil before planting. The urea was however applied separately and in solution form [18], using two split applications; first at 2 weeks after planting (WAP) and secondly at 6 WAP.

### 2.4 Soil chemical properties

A composite soil sample of about 300–500 g, was taken from the field from which the potting soil was collected. It was collected for the purpose of undergoing soil chemical analysis. This was needed to identify the nutrients that were deficient in the potting soil, prior to planting. The soil was taken from a 0–20 cm depth from the soil surface. It was analysed for organic carbon (OC), soil reaction (pH), total N, available P, available K, exchangeable calcium (Ca), exchangeable magnesium (Mg), available sulphur (S), extractable zinc (Zn), copper (Cu) and iron (Fe). The soils texture was also determined. The soil analysis procedures were carried out as follows: OC using the Walkley and Black method; pH in H_2_O using a 1:1 soil to water ratio; N was determined by the micro-Kjeldahl digestion; P by the Bray No. 1 method; sulphate-S using calcium phosphate (Ca(H_2_PO_4_)_2_) extracting solution; K, Ca and Mg using 1N ammonium acetate (NH_4_OAc) buffered at pH 7; extractable Zn, Cu and Fe using diethylenetriaminepentaacetic acid (DTPA); and soil texture using the hydrometer method [19]. The results of the soil chemical analysis are shown in Table 2, together with the ratings for how suitable each measured parameter was for cassava production. The soil was a loamy sand (85.05% sand, 11.46% clay, 2.49% silt) [34].

**Table 2:** Soil chemical characteristics of the potting soil.

Parameter	Value	Rating‡	Sufficiency range or critical level	Reference
pH	5.80	m	4.5–7.0	[21]
OC (%)	0.35	vl	4.0–10.0	[35]
N (%)	0.06	vl	0.20–0.50	[35]
P (mg/kg)	3.54	l	<4.2	[36]
K (cmol+/kg)	0.14	l	0.15–0.25	[21]
Ca (cmol+/kg)	3.04	m	1.0–5.0	[21]
Mg (cmol+/kg)	0.08	vl	0.40–1.00	[21]
S (mg/kg)	1.27	l	<6.0	[35]
Zn (mg/kg)	0.82	l	1.0–3.0	[37]
Cu (mg/kg)	0.70	m	0.3–0.8	[37]
Fe (mg/kg)	25.12	vh	4.0–6.0	[37]

‡ vl, l, m and vh stand for very low, low, medium and very high.

### 2.5 Plant management

The potting soil was deficient in N, P and K (Table 2). Clear responses were hence expected from N, P and K fertiliser application. The potting soil was additionally deficient in the nutrients Mg, S and Zn. These nutrient deficiencies were all corrected, in order to eliminate any limitations on the uptake of N, P and K, due to their deficiency in the soil. Deficiencies in Mg and S were corrected using magnesium sulphate (MgSO_4_.7H_2_O); it was applied to all pots before planting, at a rate of 25 mg Mg/kg (simultaneously adding 32.5 mg S/kg). A 1% solution of a Zn foliar fertiliser, called YaraVita Zintrac (700 g Zn/L of a ZnO foliar solution), was used to correct the Zn deficiency. Like Mg and S, Zn was also applied to all pots in the experiment. Zinc was however applied at 1 month after planting (MAP) and again at 2 MAP. A broad spectrum insecticide called Dursban (C_9_H_11_C_l3_NO_3_PS) was always mixed with the Zn foliar solution before its application.

Tap water was used to irrigate the cassava plants throughout the entire experiment. The tap water was sampled periodically. The electrical conductivity (EC_w_) of the tap water was on average 0.007 dS/m, while its pH was on average 6.58. The tap water had an average nitrate-nitrogen (NO_3_–N) content of 5.60 mg/L, with only trace amounts of phosphate-phosphorous (PO_4_–P). The tap water, additionally on average contained 0.01, 0.16, 0.25 and 0.03 meq/L of K, Na, Ca and Mg, respectively. All measured parameters were within permissible levels required for irrigation water [38, 39]. There were particularly only negligible amounts of N, P and K in the tap water, making additions of N, P and K through irrigation, negligible. The average minimum and maximum temperatures in the screen-house were 23 and 33 °C, respectively.

### 2.6 Data collected

#### 2.6.1 Shoot mass

Each cassava plant had its shoot (above-ground portion) cut-off at 1 cm above the soil surface [40]. The shoots were then individually placed in well labelled paper bags and left to dry in a screen-house for 7 days; until the oven was free to use. The paper bags were left open to allow air to freely flow in and out of them. After 7 days, the shoots, still in their paper bags, were placed in a forced draft oven, where they were left to dry for about 48 h, at 70 °C, until they attained a constant mass [37]. The shoots were then individually weighed to determine their shoot mass (g/plant) on a dry weight (dw) basis.

#### 2.6.2 New leaf formation

At the beginning of the water stress treatments (71 DAP), the position of the first fully expanded leaf from the top of each plant, was marked using a marker pen. The number of young leaves (including the unfolded leaves) above the first fully expanded leaf on each plant, was then counted and recorded. This was done to make the identification of newly formed leaves easier. The total number of leaves on each plant above the marked point, was again counted and recorded at the end of the water stress period (90 DAP). If still attached to the plant after 90 DAP, the number of young leaves initially above the marked position of the first fully expanded leaf on each plant, was subtracted from the total number of leaves above the marked position at 90 DAP. This was done to determine the total number of newly formed leaves during the 20 days of water stress. The number of leaves and/or leaf scars above the marked point, was counted to help to fully account for the young leaves that were initially on each plant before the commencement of water stress. The remaining leaves were the new leaves formed during the water stress period.

#### 2.6.3 Leaf size

Changes in the width and length of the central lobe of a cassava leaf, were used to indicate changes in leaf size. Leaf size was determined after 90 DAP, by measuring the width and length of a central leaf lobe of a fully expanded leaf, taken from a mid-height position of each plant [41]. A 15 cm ruler was used for this purpose. The mid-height position of plants was not necessarily taken as half the height of a plant, but as half the height of the remaining leaf canopy, on each plant. Measuring leaf size was however not possible on plants that had shed-off almost all of their leaves.

### 2.7 Statistical analysis

The data collected were analysed using the Analysis of Variance (ANOVA) and the Tukey's test was used to carry out mean separation at the 5% probability level [42]. All statistical analysis were carried out using GenStat Edition 14.

## 3 Results and discussion

Table 3 shows the F-test probability values obtained for the three-way ANOVA, which was carried out to assess the effects of variety, water stress and fertiliser application, on various growth characteristics of cassava, in the pot experiment [43]. The results of the ANOVA show that the dry shoot mass of cassava was influenced by the applied treatments. New leaf formation and leaf size were likewise influenced. The results of the three-way ANOVA are discussed in greater detail in the sections that follow.

**Table 3:** F-test probability values for the three-way ANOVA on the effects of variety, water stress and fertiliser application, on various growth characteristics of cassava, in the pot experiment.

Factor	Shoot mass	New formed leaves	Lobe width	Lobe length
	p-value	p-value	p-value	p-value
Variety (V)	0.002 ∗∗	0.015 ∗	<0.001 ∗∗∗	<0.001 ∗∗∗
Water stress (W)	<0.001 ∗∗∗	<0.001 ∗∗∗	0.001 ∗∗	<0.001 ∗∗∗
Fertiliser (F)	<0.001 ∗∗∗	<0.001 ∗∗∗	<0.001 ∗∗∗	<0.001 ∗∗∗
V×W	<0.016 ∗	0.321 NS	0.014 ∗	0.015 ∗
V×F	0.441 NS	0.104 NS	<0.001 ∗∗∗	0.693 NS
W×F	<0.001 ∗∗∗	0.267 NS	0.126 NS	0.285 NS
V×W×F	0.151 NS	0.859 NS	0.023 ∗	0.017 ∗

∗∗∗ Significant at p < 0.001, ∗∗ significant at p < 0.010, ∗ significant at p < 0.050 and NS not significant (p > 0.050).

### 3.1 Effects on shoot mass

**Figure 1:**
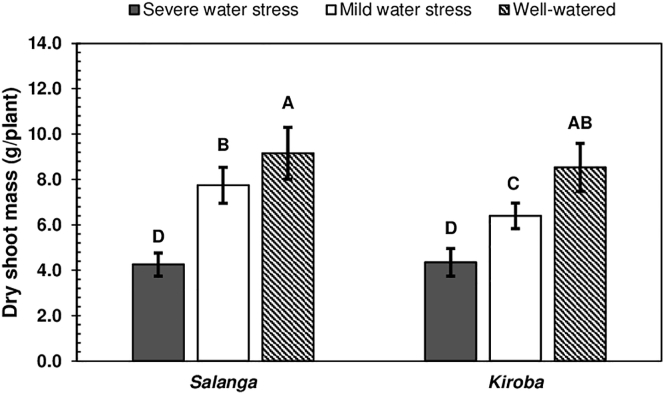
Dry shoot masses of *Salanga* and *Kiroba*, as influenced by water stress. Means (±SE) followed by the same uppercase letter are not significantly different (Tukey's test, p < 0.050). SE is the standard error of the mean.

The dry shoot masses of *Salanga* and *Kiroba*, were differently influenced by water stress (V×W, p < 0.050) (Table 3) and this was in spite of them increasing with alleviated water stress (Figure 1). The dry shoot masses of both varieties were similar under severe water stress and well-watered conditions (Figure 1). The dry shoot mass of *Salanga*, was however higher (1.2 times) than that of *Kiroba*, under mild water stress conditions. *Salanga* was hence better adapted to mild water stress, than *Kiroba*. Severe water stress, generally reduced the dry shoot masses of both varieties, by half their mass under well-watered conditions. Reductions in cassava growth as a result of water stress, were likewise observed in another pot experiment, where drought stress was shown to reduce the dry shoot mass of cassava plants, at 151 DAP, by 2.3 times their mass, under well-watered conditions (from 16.1 to 7.0 g dw) [44]. In another study, water stress was also reported to have reduced the shoot mass (from 1072 to 344 g/plant) and the root mass (from 1150 to 204 g/plant) of cassava that was this time grown in the field, at 4 to 6 MAP [45].

**Figure 2:**
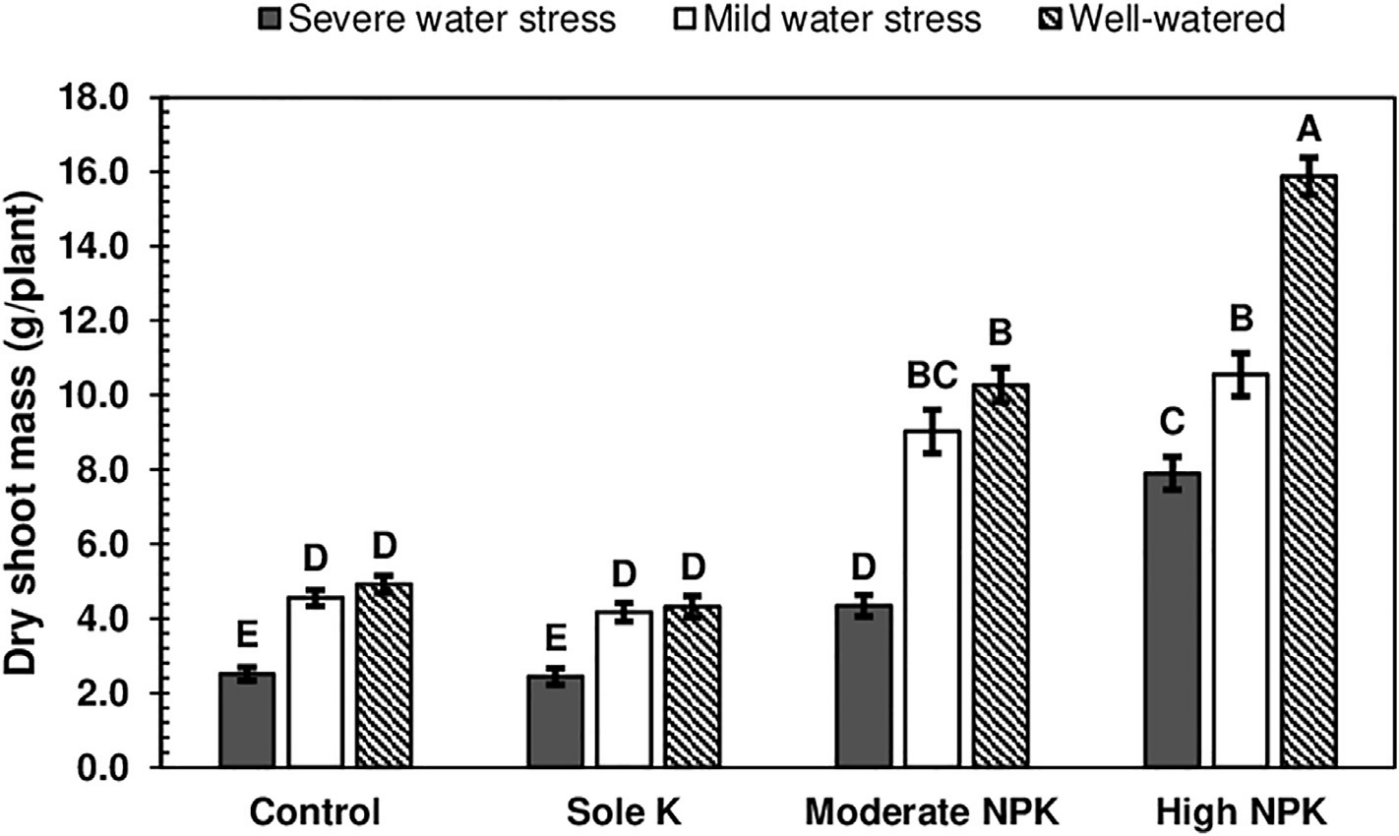
Dry shoot masses of *Salanga* and *Kiroba*, as influenced by water stress and fertiliser application. Means (±SE) followed by the same uppercase letter are not significantly different (Tukey's test, p < 0.050). SE is the standard error of the mean.

The effects of fertiliser application on the dry shoot masses of *Salanga* and *Kiroba*, was dependent on water stress (W×F, p < 0.050) (Table 3). How the dry shoot masses of *Salanga* and *Kiroba*, were both influenced by water stress and fertiliser application, is shown in Figure 2, where it can be seen that leaving plants unfertilised or supplying them with only K fertiliser, generally gave the varieties the lowest dry shoot masses, irrespective of water stress. Unfertilised cassava plants were expected to have low dry shoot masses. Higher dry shoots masses, were however expected for plants fertilised with only K, given the known benefits of K on cassava growth. Sole K fertiliser application was reported to improve the root yields (and probably the above-ground biomass) of cassava, in another study, but this was achieved with basal applications of N and P fertilisers (100 kg/ha NPK 12–12–0) [31]. The supply of N and P, was thus not limiting, in the other study, unlike in the present study. With N and P being deficient, cassava growth under the K only treatment, was thus reduced to the level of growth attained on nutrient poor soils.

Under severe water stress, the dry shoot masses of *Salanga* and *Kiroba* supplied with moderate amounts of N, P and K fertilisers, were almost twice the mass of the dry shoot masses obtained, with sole K fertiliser application and without fertiliser application (Figure 2). Moderate N, P and K fertiliser application, was therefore more beneficial for cassava growth, under severe water stress, than either sole K fertiliser application or no fertiliser application. An alleviation of severe water stress, however made *Salanga* and *Kiroba* supplied with only K fertiliser and that left unfertilised, to perform similarly as when it was supplied with moderate amounts of N, P and K fertilisers, under severe water stress. This showed that moderate N, P and K fertiliser application was just as beneficial for cassava growth, as the alleviation of severe water stress on nutrient poor soils. In another study, no differences were observed between the top biomass production of cassava supplied with moderate amounts of N, P and K fertilisers (50 kg N + 21 kg P + 41 kg K/ha) without mulching (3.18 t/ha) and that left unfertilised, but mulched (2.93 t/ha) [46]. Mulching helps to retain moisture in soils, which would otherwise be lost; it hence helps to reduce water stress. Mulching (zero water stress) without fertiliser application, therefore influenced the total biomass production of cassava, in a similar way, as fertiliser application without mulching (under water stress). In the same study, fertiliser application without mulching, however increased the root yields of cassava (5.51 t/ha), more than mulching without fertiliser application (4.66 t/ha) [46]. Moderate N, P and K fertiliser application under water stress conditions, was therefore found to be more beneficial for increasing cassava root yields, than the alleviation of water stress on nutrient poor soils.

High N, P and K fertiliser application, gave *Salanga* and *Kiroba* the highest dry shoot masses under severe water stress (Figure 2). Cassava growth, was hence better maintained with high N, P and K fertiliser application, under conditions of severe water stress. High N, P and K fertiliser application, was moreover able to effectively maintain cassava growth, across all water stress conditions. Moderate N, P and K fertiliser application only gave comparable dry shoot masses, as those attained with high N, P and K fertiliser application, under mild water stress conditions. Increments in the dry shoot masses of *Salanga* and *Kiroba*, with moderate N, P and K fertiliser application, were minimal under severe water stress, in comparison to the increments attained with high N, P and K fertiliser application. Moderate N, P and K fertiliser application was thus inconsistent at maintaining high cassava growth, across different water stress conditions. Its beneficial effects were dependent on the absence of severe water stress. This shows that cassava growth on moderately fertile soils, could at times be limited, depending on soil moisture conditions (climatic conditions).

**Figure 3:**
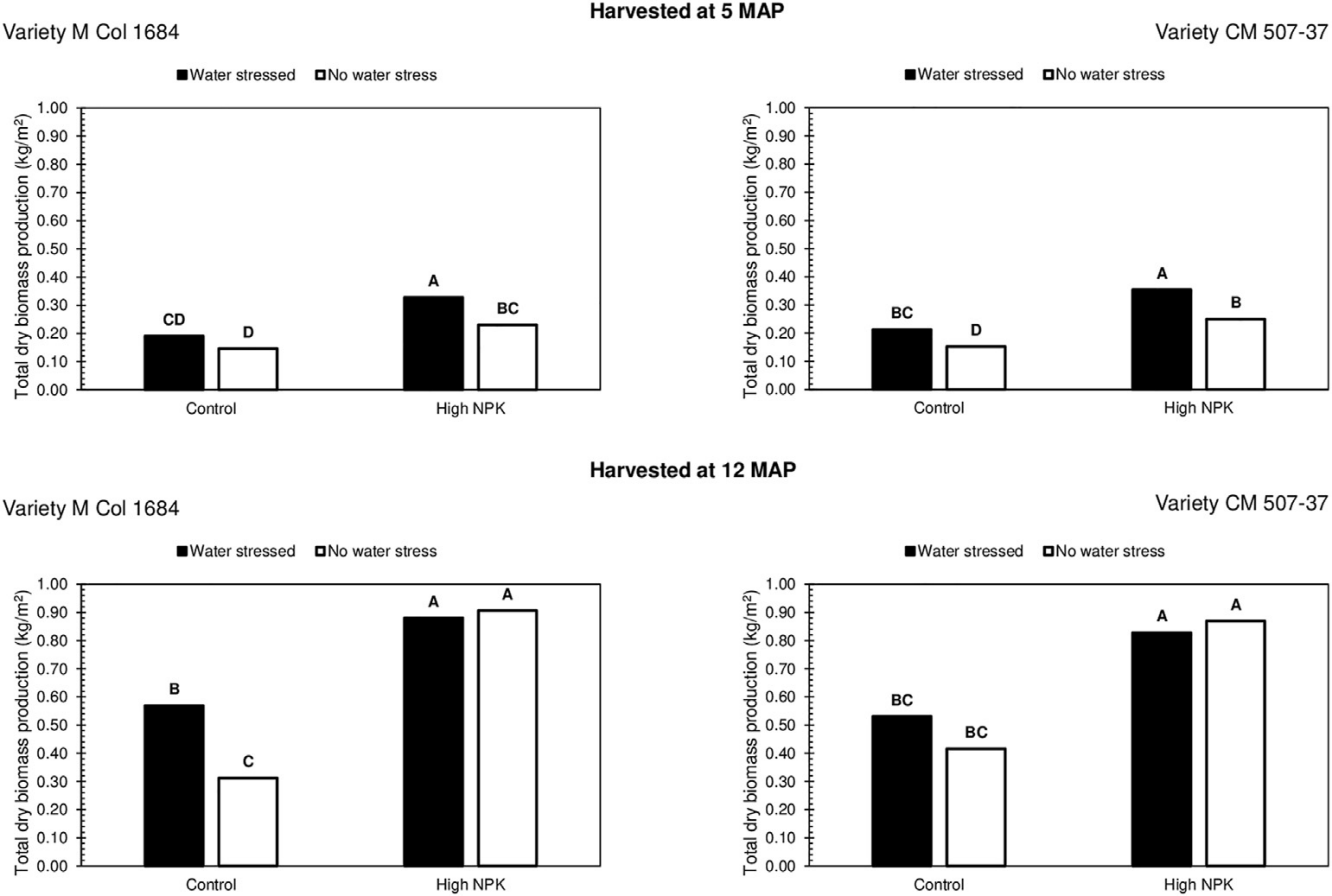
Total dry biomass production (kg/m^2^) of two field grown cassava cultivars, with and without water stress and with and without fertilisation, determined at 5 and 12 MAP (Source: De Tafur et al., 1997). Values followed by the same uppercase letter are not significantly different across both cultivars at each harvest period.

A previously mentioned study, similarly reported consistent increments of total dry biomass production (stems, leaves and roots), with high applications of N, P and K fertilisers, to cassava, irrespective of water stress [13]. This observation was made on two field grown cassava varieties at 3, 5, 7 and 12 MAP. The N, P and K fertilisers were applied at rates of 5.0 g N + 4.4 g P + 8.3 g K/m^2^ (50 kg N + 44 kg P + 83 kg K/ha), on soils with high organic matter, low levels of P and with moderate levels of K. Only moderate amounts of N were added, most likely because of the high organic matter in the soil; the supply of N to cassava was thus equally high. The results obtained in the other study are presented in Figure 3, for easier comparisons with the results obtained in the present study. Water stress was begun at 4 MAP, in the other study. The results presented in Figure 3, are only for plants harvested at 5 MAP (after 1 month of water stress) and at 12 MAP (after 8 months of water stress).

Unlike the present study (Figure 2), the alleviation of water stress in the other study, mainly resulted in decreased cassava growth (Figure 3) [13]. The effects on cassava growth that were observed in the other study, are typical of plants grown under conditions where water availability is not a limiting factor to plant growth. This normally occurs with plants or varieties that are well-adapted to water (drought) stress. Although this trend was not observed with *Salanga* and *Kiroba*, at least during their early growth and under pot conditions, both varieties are also well-adapted to water stress. The results of the other study, confirms the view that highlights that higher crop yields, on fields of low fertility, in water stressed environments, are best achieved with the use of low plant populations [8]. Low plant populations on fields of low fertility, are thought to reduce crop water use during periods of water stress; thereby maintaining crop productivity. The plant population of 1 plant per m^2^ (1 m^2^ × 1 m^2^ plant spacing) that was used in the other study, was probably more suitable for cassava growth on nutrient poor soils, under water stress conditions, hence the higher total biomass production of cassava under water stress. With fertiliser application, the total biomass production of cassava under well-watered conditions, however rose to levels similar to those obtained under water stress (Figure 3). The cassava varieties in the other study, were hence only able to effectively utilise the water supplied to them, under well-watered conditions, when supplied with adequate nutrients. Nutrient supply was hence more limiting than water supply. It must however be pointed out that this trend was more pronounced when the cassava was more mature (Figure 3).

The results of the present study (Figure 2) and of the study presented in Figure 3, are important to discuss, as they both show how cassava growth on nutrient poor soils can be influenced by the application of N, P and K fertilisers, under water stress conditions. From both studies, it is clear to see that high N, P and K fertiliser application, is essential for maintaining cassava growth on nutrient poor soils, under different water stress conditions. The yields of pearl millet (*Pennisetum glaucum* (L.) R. Br.), a crop whose productivity is equally unlimited by water availability, was similarly counteracted by the application of N and P fertilisers, in the drought-prone West African Sahel [47]. The increase in yields was observed even with increased plant populations and it remained consistent under different water stress conditions. Although fertiliser application had increased crop water use, this had not reduced the yields of pearl millet. The importance of N, P and K fertiliser application, for maintaining crop growth on nutrient poor soils, under water limited conditions, was hence once again shown.

### 3.2 Effects on new leaf formation

New leaf formation was found to be influenced by variety (V, p < 0.050), water stress (W, p < 0.050) and fertiliser application (F, p < 0.050) (Table 3). How the treatments influenced new leaf formation, is shown in Table 4, where it can be seen that *Salanga* had produced 0.6 fewer new leaves than *Kiroba*, during the water stress period. Although the difference in the number of new leaves formed between the two cassava varieties, appeared to be minimal, this was however normal, as new leaf formation rates have little genetic variation [48].

**Table 4:** New leaf formation in both *Salanga* and *Kiroba*, as influenced by variety, water stress and fertiliser application.

Factor	Treatment	Number of new leaves formed	
		Mean	SE
Variety (V)⁑	*Salanga*	5.6^b^	0.2
	*Kiroba*	6.2^a^	0.2
Water (W)	Severe water stress	4.6^b^	0.3
	Mild water stress	6.5^a^	0.2
	Well-watered	6.6^a^	0.1
Fertiliser (F)	Control	5.7^b^	0.3
	Sole K	5.6^b^	0.3
	Moderate NPK	5.4^b^	0.3
	High NPK	6.9^a^	0.3

SE is the standard error of the mean. Means followed by the same letter are not significantly different (Tukey's test, p < 0.050). ⁑Tukey's test not performed on variety means (less than three means), but letters have been added for easy interpretation.

From Table 4, it can also be seen that new leaf formation was reduced by severe water stress, but not by mild water stress. The number of new leaves formed under mild water stress, was moreover just as high as the number of new leaves formed under well-watered conditions. New leaf formation, in both *Salanga* and *Kiroba*, was therefore most affected by severe water stress. Although drought stress had equally decreased the number of leaves on cassava, in another pot experiment, different numbers of leaves were in contrast found on plants subjected to: light drought, moderate drought, severe drought and well-watered conditions [49]. The total number of leaves on cassava plants, at 151 DAP, was also lowered by drought stress (from 15.7 to 8.0 leaves per plant), in another pot experiment [44].

Table 4 also shows that the greatest increase in the number of new leaves formed, in both varieties, was brought about with high N, P and K fertiliser application. Similar results were also observed in another study, where new leaf formation rates were reported to have increased from 0.45 to 0.52 nodes/plant/day, at about 200 DAP, after an increased supply of N to cassava [50]. From Table 4, it can also be seen that all fertiliser treatments, with the exception of the high N, P and K fertiliser treatment, produced similar numbers of new leaves on plants. There was therefore no difference in the number of new leaves formed without fertiliser application and with moderate N, P and K fertiliser application, during the water stress period. No changes in the number of new leaves formed, were similarly observed in cassava, at less than 4 MAP, with moderate application of NPK fertiliser (200 kg/ha NPKMg 12:12:17:2); significant changes were however observed at more than 4 MAP [51]. The effects of moderate N, P and K fertiliser application on new leaf formation, could thus be additionally dependent on plant age. One study further found that new leaf formation was positively correlated (r = 0.4, p < 0.010) to cassava root yields [52]. High new leaf formation rates, are thus important for maintaining growth and for the attainment of high root yields in cassava.

### 3.3 Effects on leaf size (lobe widths and lengths)

There was a three-way interaction effect of variety, water stress and fertiliser application (V×W×F, p < 0.050), on the lobe widths and lobe lengths of cassava leaves (Table 3). The significant three-way interaction effect revealed that the lobe widths and lobe lengths of each cassava variety, were differently influenced by water stress and fertiliser application. The data was therefore split by variety and separate two-way ANOVAs were carried out, to find out how each variety was differently influenced by water stress and fertiliser application. The F-test probability values obtained for the two-way ANOVAs, are shown in Table 5.

**Table 5:** F-test probability values for the two-way ANOVA on the effects of water stress and fertiliser application, on the lobe widths and lobe lengths of *Salanga* and *Kiroba* leaves.

Factor	*Salanga*		*Kiroba*	
	Lobe width	Lobe length	Lobe width	Lobe length
	p-value	p-value	p-value	p-value
Water stress (W)	0.359 NS	0.085 NS	0.004 ∗∗	<0.001 ∗∗∗
Fertiliser (F)	0.595 NS	<0.001 ∗∗∗	<0.001 ∗∗∗	<0.001 ∗∗∗
W×F	0.530 NS	0.457 NS	0.049 ∗	0.003 ∗∗

∗∗∗ Significant at p < 0.001, ∗∗ significant at p < 0.010, ∗ significant at p < 0.050 and NS not significant (p > 0.050).

Table 5 shows that the lobe widths of *Salanga* leaves were unaffected by both water stress (W, p > 0.050) and fertiliser application (F, p > 0.050). They hence remained unchanged with varying levels of water stress and with changes in the combination and amount of fertiliser applied (Table 6). The lobe widths of *Salanga* (mean = 0.9 cm, SE = 0.0), were generally 2.3 times narrower than those of *Kiroba* (mean = 2.0 cm, SE = 0.1). Narrow lobe widths appeared to be an inherent characteristic of the leaves of *Salanga*. Central lobe widths for the leaves of various cassava varieties, can range from 1 to 6 cm [53]. The lobe widths of *Salanga* leaves, were hence revealed to be amongst the narrowest lobe widths, of all cassava varieties.

**Table 6:** Lobe widths and lobe lengths of *Salanga* leaves, as influenced by water stress and fertiliser application.

Factor	Treatment	Lobe width		Lobe length	
		Mean⁑	SE	Mean	SE
		(cm)	(cm)	(cm)	(cm)
Water stress (W)	Severe water stress	0.8	0.0	10.0	0.3
	Mild water stress	0.9	0.0	10.6	0.3
	Well-watered	0.9	0.0	10.1	0.3
Fertiliser (F)	Control	0.9	0.0	9.3^c^	0.2
	Sole K	0.8	0.0	9.3^c^	0.1
	Moderate NPK	0.8	0.0	10.4^b^	0.3
	High NPK	0.9	0.0	11.9^a^	0.3

SE is the standard error of the mean. Means followed by the same letter are not significantly different (Tukey's test, p < 0.050). ⁑Means with no letter beside them are not significantly different (p > 0.050), mean separation was thus not performed.

In contrast to its lobe widths, the lobe lengths of *Salanga* leaves were at least influenced by fertiliser application (F, p < 0.050) (Table 5). The lobe lengths of *Salanga* obtained with moderate N, P and K fertiliser application, were 1.1 times shorter than the lobe lengths obtained with high N, P and K fertiliser application (Table 6). The lobe lengths obtained when *Salanga* was unfertilised or when it was supplied with only K, were on the other hand, 1.3 times shorter than the lobe lengths obtained with high N, P and K fertiliser application. The greatest increase in the size of *Salanga* leaves, was therefore obtained with high N, P and K fertiliser application and this was irrespective of water stress. An increase (of 17%) in the lobe lengths of cassava leaves, was similarly reported, in another study, with an improved supply of N [50]. A low supply of P and particularly of N, in the control and sole K treatments, probably led to the restricted growth of lobe lengths under the two treatments. The lobe lengths of *Salanga* (mean = 10.2 cm, SE = 0.2) were generally 1.4 times longer than those of *Kiroba* (mean = 7.4 cm, SE = 0.2). The growth of *Salanga* leaves appeared to be dependent on changes in its lobe lengths; this must have compensated for its permanently narrow lobe widths. The central lobe lengths, for the leaves of various cassava varieties, can range from 4 to 20 cm [53]. The lobe lengths of *Salanga* leaves, were thus only moderately as long as those of other cassava varieties.

**Figure 4:**
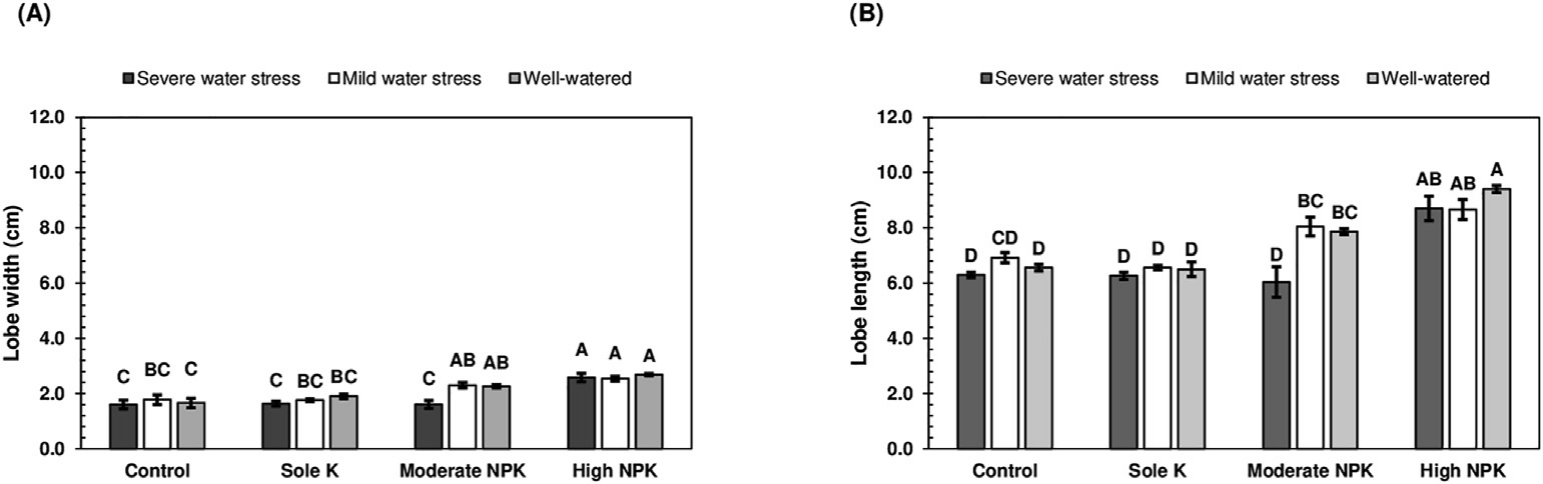
Lobe widths (A) and lobe lengths (B) of *Kiroba* leaves, as influenced by water stress and fertiliser application. Means (±SE) followed by the same uppercase letter are not significantly different (Tukey's test, p < 0.050). SE is the standard error of the mean.

Unlike *Salanga*, both the lobe widths and lobe lengths of *Kiroba* leaves, were influenced by water stress and fertiliser application. The effects of fertiliser application were however dependent on water stress (W×F, p < 0.050) (Table 5). Water stress and fertiliser application moreover influenced the lobe widths and lobe lengths of *Kiroba*, in a similar manner. From Figure 4, it can be seen that except when supplied with moderate amounts of N, P and K fertilisers, the lobe widths and lobe lengths of *Kiroba* leaves, remained unchanged, regardless of water stress conditions. Water stress was therefore only able to influence the lobe widths and lobe lengths of *Kiroba*, when it was supplied with moderate amounts of N, P and K fertilisers. With moderate N, P and K fertiliser application, the lobe widths and lobe lengths of *Kiroba* leaves, were respectively narrower and shorter under severe water stress, but respectively wider and longer, under both mild water stress and well-watered conditions. Other studies have also reported reductions in the size of cassava leaves after drought stress [54, 55]. *Kiroba* leaves were the smallest with moderate N, P and K fertiliser application, under severe stress and also when *Kiroba* was left unfertilised or supplied with only K fertiliser, regardless of water stress. Moderate N, P and K fertiliser application, was only able to increase the lobe widths and lobe lengths of *Kiroba* leaves, after the alleviation of severe water stress.

The lobe widths and lobe lengths of *Kiroba* leaves obtained with moderate N, P and K fertiliser application under mild water stress, were similar to those obtained with high N, P and K fertiliser application under severe water stress (Figure 4). High N, P and K fertiliser application, hence produced a greater increase in the size of *Kiroba* leaves, under severe water stress. Both moderate and high N, P and K fertiliser application, however produced similar lobe widths and lobe lengths, in *Kiroba*, under mild water stress conditions (Figure 4). The two fertiliser rates were thus equally effective at increasing the lobe widths and lobe lengths of *Kiroba* leaves, under mild water stress conditions. High N, P and K fertiliser application, however brought about the most consistent increase in the size of *Kiroba* leaves, regardless of water stress conditions.

## 4 Conclusions

The study managed to demonstrate that when applied on nutrient poor soils, N, P and K fertiliser application, can help to maintain relatively high cassava growth, across different water stress conditions. The study however found that high and not moderate N, P and K fertiliser application, was required to consistently achieve relatively high cassava growth, across all water stress conditions. Moderate N, P and K fertiliser application, was only as effective under mild water stress conditions. Following the findings, only cassava cultivated in environments that never experience severe water stress, may hence benefit from moderate N, P and K fertiliser application. Environments prone to severe water stress or indeed to both severe and mild water stress, would in contrast benefit more from high N, P and K fertiliser application.

Larger leaf sizes and the formation of higher numbers of new leaves, on cassava plants, was what led to the relatively higher growth, achieved with high N, P and K fertiliser application, under water stress conditions. Although the study did not account for the number of leaves shed by the cassava plants, it can be assumed that relatively larger leaf canopy sizes, were needed to maintain adequate cassava growth, during the water stress period. Large leaf canopies, however contribute to water loss through increased transpiration. The relatively high dry matter production that occurs with high N, P and K fertiliser application, for every unit of water supplied under water stress conditions, would however help to compensate for any water loss. High N, P and K fertiliser application, therefore gives cassava a high WUE, enabling it to maintain relatively high productivity, under water stress conditions. The findings from the pot experiment, are however only indicative of how cassava growth on nutrient poor soils, would be influenced by N, P and K fertiliser application, across different water stress conditions. The findings therefore need to be verified under field conditions.

## Declarations

### Author contribution statement

Matema L.E. Imakumbili: Conceived and designed the experiments; Performed the experiments; Analyzed and interpreted the data; Contributed reagents, materials, analysis tools or data; Wrote the paper.

Ernest Semu: Conceived and designed the experiments.

Johnson M.R. Semoka: Conceived and designed the experiments; Contributed reagents, materials, analysis tools or data.

Adebayo Abass; Geoffrey Mkamilo: Contributed reagents, materials, analysis tools or data.

### Funding statement

This work was supported by the 10.13039/100013718Alliance for a Green Revolution in Africa (AGRA) (2009 SHP 027) and by the 10.13039/100000865Bill and Melinda Gates Foundation (OPP48790).

### Data availability statement

Data associated with this study has been deposited at Mendeley Data under doi: 10.17632/J3FHN7P9KX.1.

### Declaration of interests statement

The authors declare no conflict of interest.

### Additional information

No additional information is available for this paper.
